# MicroRNA Expression Is Altered in an Ovalbumin-Induced Asthma Model and Targeting miR-155 with Antagomirs Reveals Cellular Specificity

**DOI:** 10.1371/journal.pone.0144810

**Published:** 2015-12-22

**Authors:** Maximilian W. Plank, Steven Maltby, Hock L. Tay, Jessica Stewart, Fiona Eyers, Philip M. Hansbro, Paul S. Foster

**Affiliations:** Centre for Asthma and Respiratory Diseases, School of Biomedical Sciences and Pharmacy, Faculty of Health, University of Newcastle and Hunter Medical Research Institute, Callaghan, NSW 2300, Australia; IRCCS-Policlinico San Donato, ITALY

## Abstract

MicroRNAs are post-transcriptional regulators of gene expression that are differentially regulated during development and in inflammatory diseases. A role for miRNAs in allergic asthma is emerging and further investigation is required to determine whether they may serve as potential therapeutic targets. We profiled miRNA expression in murine lungs from an ovalbumin-induced allergic airways disease model, and compared expression to animals receiving dexamethasone treatment and non-allergic controls. Our analysis identified 29 miRNAs that were significantly altered during allergic inflammation. Target prediction analysis revealed novel genes with altered expression in allergic airways disease and suggests synergistic miRNA regulation of target mRNAs. To assess the impacts of one induced miRNA on pathology, we targeted miR-155-5p using a specific antagomir. Antagomir administration successfully reduced miR-155-5p expression with high specificity, but failed to alter the disease phenotype. Interestingly, further investigation revealed that antagomir delivery has variable efficacy across different immune cell types, effectively targeting myeloid cell populations, but exhibiting poor uptake in lymphocytes. Our findings demonstrate that antagomir-based targeting of miRNA function in the lung is highly specific, but highlights cell-specificity as a key limitation to be considered for antagomir-based strategies as therapeutics.

## Introduction

Allergic asthma is characterized by immune hypersensitivity to allergens [1]. The mechanisms that lead to hypersensitivity are complex and include the activation of endothelial and immune cells, immune cell recruitment and changes in gene expression [1]. Despite our knowledge of factors influencing disease pathogenesis, the available treatments for asthma and allergic inflammation remain insufficient to adequately control disease [2]. As such, novel therapeutic strategies are required for the effective treatment of asthma and allergic inflammation.

MicroRNAs (miRNAs) are conserved single-stranded RNA molecules (around 22 nucleotides in length) that regulate gene expression by targeting the 3’UTR of mRNA transcripts [3]. MiRNA targeting inhibits translation and destabilizes target mRNAs, resulting in reduced protein production. MiRNAs play important roles in a variety of biological processes, including apoptosis, cancer, development and inflammation [4–6]. MiRNA inhibitors are emerging as effective therapeutics in a range of diseases, including cancer, hepatitis and metabolic disorders [7, 8]. MiR-122, in particular, is a promising target for the treatment of hepatitis C, with human clinical trials currently under way [8].

To date, few studies have investigated the role of miRNAs in asthma and murine models of allergic airways disease (AAD). Several recent human studies provide evidence that miRNAs may be involved in asthmatic disease [9–11]. In mouse models of AAD, important functions for let-7, miR-21, miR-126, miR-145 and miR-155 have been demonstrated in disease pathogenesis [12–19]. MiR-21 expression was increased in several models of AAD and mir-21 deficient mice have reduced Th2 responses and decreased eosinophilia in an ovalbumin (OVA)-induced model of AAD [12, 13]. Our group identified upregulation of miR-126 in a house dust mite (HDM)-induced AAD model [16, 20]. Localised inhibition of miR-126 in the lung with an antagomir (antisense miRNA inhibitor) significantly reduced hallmark features of AAD [16]. Our group also observed that miRNAs let-7b, miR-21 and miR-145 were upregulated in a HDM-induced model of AAD [17]. While antagomir administration could specifically reduce the levels of each individual miRNA, inhibition of let-7b and miR-21 had no significant effect on disease pathogenesis [17]. However, blockade of miR-145 did markedly reduce hallmark features of disease [17]. Mir-155^-/-^ mice which lack pri-mir-155 and mature miRNAs, miR-155-5p and miR-155-3p, also have decreased lung inflammation in models of AAD (18, 19). These findings clearly demonstrate that individual miRNAs are altered and play functional roles in AAD.

In the present study, we performed miRNA array experiments to reveal altered miRNA expression (of 29 miRNAs) in an acute, steroid-sensitive, OVA-driven murine model of AAD. Bioinformatics analysis predicted novel mRNA targets that could be synergistically regulated by the induced miRNAs, which we confirmed at the mRNA level by quantitative PCR (qPCR). *In vivo* targeting of one induced miRNA (mmu-miR-155-5p; referred to as miR-155-5p) with a specific antagomir successfully reduced miR-155-5p levels, but failed to alter disease pathogenesis. Further analysis revealed differing efficacy of antagomir-mediated targeting across different immune cell types both *in vitro* and *in vivo*. Specifically, antagomir uptake was highly efficient in myeloid cell populations, but was ineffective at targeting T lymphocytes. Our findings clearly demonstrate altered miRNA gene expression and revealed novel changes in gene expression during AAD. Importantly, we also demonstrate variable cell targeting as a key limitation of antagomir-based strategies, which must be considered when interpreting findings from antagomir studies and when designing therapeutic interventions.

## Materials and Methods

### Ethics statement

This study was carried out in strict accordance with the requirements of the Australian Code of Practice for the Care and Use of Animals for Scientific Purposes. The protocol was specifically approved by the Animal Care and Ethics Committee (ACEC) of the University of Newcastle (Permit numbers #1060 and #A-2010-148).

### Mice and animal welfare

Specific pathogen-free (SPF) BALB/c mice (8–12 weeks old) were obtained from the University of Newcastle’s SPF facility and housed in individually ventilated cages in approved containment facilities within the Bioresources Facility, Hunter Medical Research Institute (Newcastle, Australia). Mice were provided with water and food *ad libitum* and acclimatised for one week prior to experimentation. All efforts were made to minimize suffering in treated animals. All treated animals were monitored daily as part of the approved protocol for weight loss and clinical score. Clinical scores were defined as: 0 = no clinical signs, 1 = rough coat, 2 = rough coat, less reactive, passive during handling. Intervention points by euthanasia were set as weight loss of over 20% body weight or reaching a clinical score of 2. There were no animal deaths or interventions required as a result of our protocol. All mice were euthanised by pentobarbital overdose (Virbac, Australia).

### Murine model of allergic airways disease

AAD was induced through sensitisation and inhalational challenges as previously described [21]. Briefly, mice were systemically sensitised via i.p. injection of 50 μg chicken egg OVA (Grade V, ≥98% pure, Sigma, Australia) complexed with 1 mg Alhydrogel (Sigma, Australia) in saline (day 0). Non-sensitized control mice were injected with 1 mg Alhydrogel in saline. On days 12, 13, 14 and 15, all mice were challenged (i.n.) with 10 μg of OVA in saline. Endpoints were performed 24 hours after the last OVA challenge. Antagomir treatments (50 μg/dose of Ant i.n.) were performed on days 11, 13 and 15, 6 hours before challenges. Dexamethasone (DEX) treatments (3 mg/kg body weight, i.p.) were performed on day 11 and every second day thereafter 1 hour before challenges. Intranasal treatments of animals were performed under light anesthesia with isoflurane (Pharmachem, Australia).

### Airway hyper-responsiveness (AHR) measurement

AHR was assessed invasively in mice anesthetised with 100 mg/kg ketamine (Ceva, Australia) and 10 mg/kg xylazine (Ilium, Australia) by measurement of total lung resistance and dynamic compliance using a Finepoint^TM^ series Resistance and Compliance system (Buxco, North Carolina USA) [22]. Percentage increase over baseline (PBS) in response to increasing doses of nebulized methacholine was calculated.

### Collection and analysis of bronchoalveolar lavage (BAL) fluid

The left lobe of the lung was tied off, leaving the right lung open for BAL fluid collection. BAL samples were collected in Hanks Buffered Saline Solution (HBSS) (Gibco, Invitrogen) using a cannula inserted into the trachea. For differential cell counts, BAL samples were prepared by centrifugation, red blood cell lysis in hypotonic solution, counted on a hematocytometer and cytospun onto glass slides (Shandon-4). Cytospin slide preparations were stained with May Grünwald and Giemsa stain according to manufacturer’s instructions (ACR, Australian Scientific). Slides were analysed by light microscopy and differential cell counts performed based on cell morphology (minimum of 300 cells per slide).

### Lung histology

A transverse section of the left lung lobe was prepared and fixed in 10% neutral buffered formalin and processed for histology (John Curtin School of Medical Research, Australian National University, Canberra, ACT, Australia). Fixed tissue was embedded in paraffin and sections were stained with either hematoxylin and eosin (for eosinophil quantification) or Periodic acid-Schiff (for mucous secreting cell quantification). Sections were examined by light microscopy. Cell counts were performed and averaged to give a result in cells/100 μm.

### RNA extraction

Total RNA samples were purified from lung tissue (either whole lung or blunt dissected airway and parenchyma) for miRNA profiling and gene and miRNA expression analysis. Lung tissue was placed in RNALater (Invitrogen, Carlsbad, CA) and stored at -80˚C. Total RNA was isolated by phenol-chloroform separation and isopropanol precipitation, quantified on a Nanodrop 1000 spectrophotometer (Thermo Scientific, USA) and assessed using an 2100 Bioanalyzer Instrument (Agilent Technologies, USA). Samples with a RNA integrity number (RIN) below 8 were excluded from further analysis.

### MiRNA microarrays

MiRNA microarrays were performed according to manufacturer’s protocol using the miRNA Complete Labeling and Hyb Kit and Mouse miRNA Microarray Release 12.0 slides (Agilent Technologies, Technology number 21828, version 2010.11.04). Samples were hybridized, washed and scanned with a High-Resolution C Scanner (Agilent Technologies, USA). Data was processed using the Feature Extraction software version 10.7.3.1 and data analysis was performed using Genespring GX version 11.5.1 (Agilent Technologies, USA). The miRNA microarray data has been made publicly available at NCBI (GEO accession number GSE72793).

### Quantitative PCR

For gene expression analysis, qPCR was performed using random hexamer primers (Invitrogen) and Superscript III (Invitrogen), on a T100 thermal cycler (Bio-Rad, Hercules, CA). Relative qPCR quantiﬁcation was performed on a Mastercycler® ep realplex system (Eppendorf, Australia), using SYBR reagents. Primer sets (Table 1) were designed to specifially amplify mRNA products and results normalized to the house-keeping gene HPRT. For the detection of pri-mir-155, a specific primer set (Mm03306395_pri) and TaqMan® Universal PCR Master Mix No AmpErase UNG (both Life Technologies) was used for qPCR quantification.

**Table 1:** Primer sequences used for qPCR analysis (indicated in 5’-3’ orientation).

Gene	Forward primer	Reverse primer
ATXN1	GCCGTGATACAGTTTGCTGTTGGT	GGATGACCAGCCCTGTCCAAATAC
ACVR2A	GGCCAGCATCCATCTCTTGAA	TTTCACAGAGCATTGCCATTCC
HPRT	AGGCCAGACTTTGTTGGATTTGAA	CAACTTGCGCTCATCTTAGGCTTT
IL-5	TGTTGACAAGCAATGAGACGATGA	AATAGCATTTCCACAGTACCCCCA
IL-13	AGCTGAGCAACATCACACAAGACC	TGGGCTACTTCGATTTTGGTATCG
MIER3	CAATATGGCGGAGGCTTCCTTT	GAACTAGCATCTCAGCAGTGGG
MUC5AC	CGGCCGGAGAAAGTTGGTCCC	GCACACCCGCCTGGTATGTCC
NFAT5	TCAATGAGTCAGACAAGCGGTG	AGGAGGGAGCTGAAGAAGCATC
NR3C1	TGACTCTACCCTGCATGTATGACCA	CTCTTGGCTCTTCAGACCTTCCTT
PTEN	TGGATTCGACTTAGACTTGACCT	GCGGTGTCATAATGTCTCTCAG
TAB2	GGATCTGATGATGCTGCCTACA	TTGCATCTCGAGCTCTCTTTGA
TNRC6B	TACTGGCTGGTTCTCCACAATC	TCTTGTTTGGTGCTGTATCGGA

For miRNA expression analysis, miRNA-specific cDNA was generated using a TaqMan® MicroRNA Reverse Transcription Kit and gene-specific primers (mmu-miR-18a-5p (ID 002422), mmu-miR-21a-5p (ID 000397), mmu-miR-144-3p (ID 002676), mmu-miR-155-5p (ID 002571), mmu-miR-155-3 (464539_mat), mmu-miR-188-5p (ID 002320), mmu-miR-223-3p (ID 002295), mmu-miR-449a-5p (ID 001030), mmu-miR-451a (ID 001141) and snoRNA202 (ID 001232); Life Technologies). qPCR was then performed on a Mastercycler® ep realplex system (Eppendorf, Australia) using TaqMan® Universal PCR Master Mix No AmpErase UNG and normalized to the house-keeping RNA sno202.

### Antagomirs (Ant)

Target miRNA sequences from miRBase (Wellcome Trust, Sanger Institute, Cambridge, United Kingdom) were used to design specific antisense Ant. Ant-155 (5'mA.*.mC.*.mC.mC.mC.mU.mA.mU.mC.mA.mC.mA.mA.mU.mU.mA.mG.mC.mA.mU.*.mU.*.mA.*.mA.*. 3'-Chol) and a scrambled control (Scramble; 5'mU.*.mC.*.mA.mC.mA.mA.mC.mC.mU.mC.mC.mU.mA.mG.mA.mA.mA.mG.mA.*.mG.*.mU.*.mA.*.3'-Chol) were synthesized by Dharmacon Inc (Lafayette, Colorado), where m denotes 2’-O-Methyl-modified phosphoramidites, * denotes phosphorothioate linkages, and -Chol denotes hydroxyprolinol-linked cholesterol. Fluorescently labeled Ant containing a 5’-DY547 phosphoramidite modification was used where indicated.

### T cell culture

CD4^+^ T cells were purified from spleen tissue by magnetic cell separation using murine CD4 (L3T4) microbeads according to manufacturer’s protocol (Miltenyi Biotec, Germany). CD4^+^ T cells were then stained with fluorescently labeled antibodies against CD4, CD44, CD62L and CD25 and sorted on a FACSAria cell sorter (BD Pharmingen, USA) to purify naïve T cells (CD4^+^CD44^lo^CD62L^hi^CD25^-^; clones RM4-5; IM7, MEL-14, PC61; BD Pharmingen, USA). For proliferation assays, CD4^+^ T cells were labeled with carboxyfluorescein diacetate succinimidyl ester (CFSE) as previously described [23]. For the activation of Th cells, purified CD4^+^ T cells were stimulated in complete media (DMEM, 10% FCS, 2 mM L-glutamine, 50 μM β-mercaptoethanol, 100U/ml Penicillin-Streptomycin; Invitrogen, Australia) with plate-bound anti-CD3ε (1 μg/ml; Clone 145-2C11; BD Pharmingen, USA) and soluble anti-CD28 (2 μg/ml; Clone 37.51; BD Pharmingen, USA) for 3 days.

### Flow cytometry

Lung tissue was digested in HEPES buffer containing collagenase D (Sigma-Aldrich, St. Louis, MO) and DNAse, then forced through a 70-μm strainer. Spleen samples were forced through 70-μm strainers without enzymatic digestion. After isolation, red blood cell lysis was performed using ammonium chloride solution. For *in vitro* Ant uptake assays, CD4^+^ T cells were separated with magnetic beads and both CD4^+^ and CD4^-^ cell preparations were incubated with fluorescent Ant for 1 h at the indicated concentrations. For flow cytometry staining, all cells were treated with Fc block before staining with fluorochrome-conjugated Abs. For flow cytometry analysis, labeled cell samples were fixed overnight in PBS containing 2% FCS and 0.1% paraformaldehyde, collected on a BD FACSCanto II flow cytometer and analyzed using FACSDiva software (BD Biosciences). For fluorescence-activated cell sorting, fluorochrome-labeled cell preparations were separated on a FACSAria III cell sorter (BD Pharmingen, USA) based on surface marker expression, as indicated. The purity of sorted cells was confirmed as >99%. The following antibodies were used (BD Biosciences): B220 (RA3-6B2), CD3e (145-2C11), CD4 (RM4-5), CD8a (56–6.7), CD11b (M1/70), CD11c (N418), CD45 (30-F11), Epcam (G8.8), F4/80 (BM8), Ly6G (1A8), SiglecF (E50-2440).

### Statistical analysis

P values were calculated using the unpaired Mann-Whitney test. P values < 0.05 were considered statistically significant.

## Results

### MiRNA profiling identifies differential miRNA expression in AAD lung

**Fig 1:**
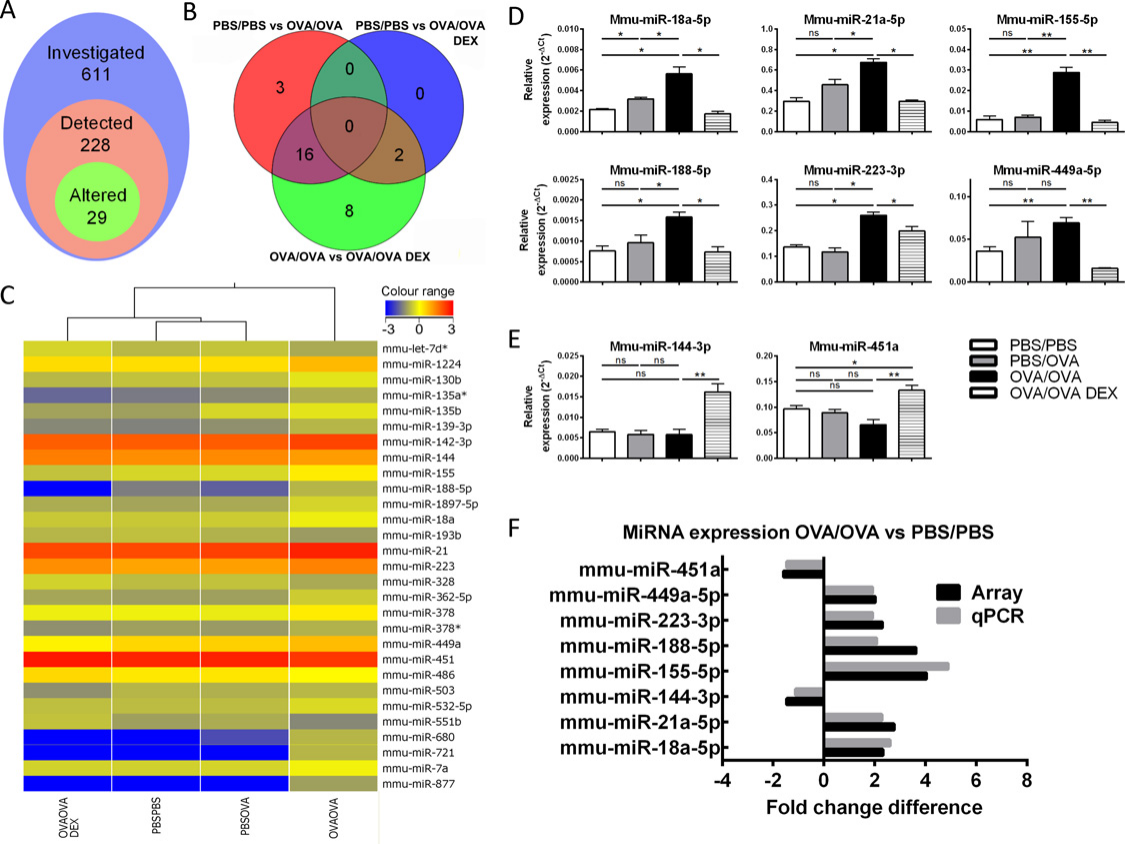
MiRNA profiling identifies differential miRNA expression in lungs of allergic mice. Expression of 611 miRNAs was investigated in whole lung samples by microarray. 228 miRNAs were detected in the lungs, with 29 altered between experimental groups (A). Venn diagram of statistically-significant differentially expressed miRNAs (fold-change ≥2) between PBS/PBS (PBS) and OVA/OVA, PBS/PBS and OVA/OVA DEX (DEX) and OVA/OVA and OVA/OVA DEX (B). Hierarchical cluster analysis of treatment conditions based on differentially expressed miRNAs. Heat-map representation of normalised expression levels for differentially expressed miRNAs. The boxes in colour indicate the normalised intensities of miRNA expression, with blue indicating low and red indicating high expression (C). qPCR detection of 6 altered miRNAs (D) and two DEX-responsive miRNAs (E). Expression is normalised relative to control RNA sno202. Fold changes for selected miRNAs is graphed for both microarray and qPCR analysis (OVA/OVA versus PBS/PBS) (F). Error bars represent SEM (n = 6 animals/group). Mann-Whitney test was used to compare two groups with another. P values of less than 0.05 are indicated by * and less than 0.01 by ** and less than 0.001 by ***.

To assess the role of miRNAs in AAD, we utilised a steroid-sensitive, OVA-driven mouse model (see S1 Fig for characterisation of inflammation and morphological changes). We performed microarray analysis of miRNA expression in whole lung samples from PBS-sensitised/challenged mice (PBS/PBS), PBS-sensitised/OVA-challenged mice (PBS/OVA), OVA-sensitised/challenged mice (OVA/OVA) and OVA-sensitised/challenged mice treated with dexamethasone (OVA/OVA DEX). 228 of the total 611 arrayed miRNAs were detected in mouse lungs in at least one experimental group (Fig 1A). Of the 228 expressed miRNAs, 29 miRNAs exhibited significant differences in expression between one or more groups (Fig 1A–1C, Table 2).

**Table 2:** Differentially expressed miRNAs in whole lung. Systematic name and fold changes for the different treatment groups are given for 29 miRNAs. Fold changes over 2 are highlighted in bold.

Systematic Name	FC PBS/OVA vs PBS/PBS	FC OVA/OVA vs PBS/PBS	FC OVA/OVA vs PBS/OVA	FC OVA/OVA vs OVA/OVA DEX	FC OVA/OVA DEX vs PBS/PBS
**mmu-let-7d-3p**	1.32	-1.38	-1.82	**-2.60**	1.89
**mmu-miR-1224-5p**	-1.02	**2.48**	**2.54**	**2.40**	1.04
**mmu-miR-130b-3p**	1.00	**2.22**	**2.21**	**2.87**	-1.30
**mmu-miR-135a-1-3p**	1.43	**3.40**	**2.37**	**5.12**	-1.51
**mmu-miR-135b-5p**	**3.53**	**4.86**	1.38	**4.51**	1.08
**mmu-miR-139-3p**	1.38	**3.25**	**2.36**	**2.81**	1.16
**mmu-miR-142-3p**	-1.02	1.90	1.93	**2.01**	-1.06
**mmu-miR-144-3p**	1.10	-1.47	-1.61	**-2.23**	1.52
**mmu-miR-155-5p**	1.01	**4.04**	**4.00**	**6.66**	-1.65
**mmu-miR-188-5p**	-1.96	**3.63**	**3.12**	**3.51**	**-2.99**
**mmu-miR-1897-5p**	1.19	**3.12**	**2.62**	**2.58**	1.21
**mmu-miR-18a-5p**	1.05	**2.33**	**2.21**	**2.39**	-1.03
**mmu-miR-193b-3p**	-1.35	**-2.29**	-1.70	-1.87	-1.22
**mmu-miR-21a-5p**	1.29	**2.77**	**2.15**	**2.64**	1.05
**mmu-miR-223-3p**	1.08	**2.30**	**2.12**	1.30	1.76
**mmu-miR-328-3p**	1.13	-1.58	-1.78	**-2.56**	1.62
**mmu-miR-362-5p**	1.07	**2.95**	**2.77**	**2.40**	1.23
**mmu-miR-378a-3p**	-1.13	**2.21**	**2.50**	**2.29**	-1.03
**mmu-miR-378a-5p**	-1.16	1.52	1.76	**2.19**	-1.45
**mmu-miR-449a-5p**	1.20	**2.02**	1.68	**4.19**	**-2.08**
**mmu-miR-451a**	1.05	-1.59	-1.68	**-2.36**	1.48
**mmu-miR-486-5p**	1.01	-1.62	-1.64	**-2.44**	1.51
**mmu-miR-503-5p**	-1.14	1.06	1.21	**2.62**	**-2.47**
**mmu-miR-532-5p**	1.02	**2.05**	**2.02**	1.94	1.06
**mmu-miR-551b-3p**	1.34	-1.72	**-2.30**	**-3.74**	**2.18**
**mmu-miR-680**	1.03	**6.24**	**6.24**	**6.24**	-1.30
**mmu-miR-721**	1.54	**6.31**	**4.61**	**6.31**	1.70
**mmu-miR-7a-5p**	1.08	**2.12**	1.97	**2.09**	1.01
**mmu-miR-877-5p**	-1.19	**5.14**	**5.14**	**5.14**	-1.42

Only one miRNA (mmu-miR-135b-5p) was detected with >2-fold change in the PBS/PBS compared to PBS/OVA group. Four miRNAs were altered >2-fold in the OVA/OVA DEX-treated group compared to PBS/PBS (mmu-miR-551b-3p increased and mmu-miR-188-5p, -449a-5p and -503-5p decreased). The majority of altered miRNAs (22 of 29) were upregulated (compared to 7 downregulated miRNAs) in the OVA/OVA allergic group compared to PBS/PBS, PBS/OVA and OVA/OVA DEX (Fig 1C, Table 1). Hierarchical clustering of the differentially expressed miRNAs demonstrated the most similarity between PBS/PBS and PBS/OVA, followed by OVA/OVA DEX (Fig 1C). The OVA/OVA group exhibited the largest differences when compared to the other experimental groups. Interestingly, most of the differentially regulated miRNAs (26 of 29) were steroid-sensitive, demonstrating alterations after DEX treatment (Fig 1B and 1C).

To verify our microarray results, we selected 8 (from the 29 altered) miRNAs and performed qPCR. The 8 miRNAs were selected based on their expression pattern, expression level and existing evidence for a role in inflammatory disease (Fig 1D and 1E). Six of the miRNAs assessed (mmu-miR-18a-5p, -21a-5p, -155-5p, 188-5p, -223-3p and -449a-5p) were significantly increased following OVA sensitization and challenge (Fig 1D). All 6 of these miRNAs decreased following DEX treatment, demonstrating steroid-sensitivity (Fig 1D). Two of the assessed miRNAs (mmu-miR-144-3p and -451a) were increased in response to DEX treatment (Fig 1E). We also assessed the expression levels of two miRNAs previously associated with lung disease (mmu-miR-146a-5p and mmu-miR-221) [24, 25], but could not detect differential expression in this study (data not shown). Importantly, qPCR-validated expression profiles closely matched our array data, confirming the validity of the observed alterations (Fig 1F). Taken together, we could successfully determine the lung miRNA expression profile and identify AAD-associated miRNA expression changes in the lung.

### Identification of novel predicted targets of altered miRNAs

To identify potential mRNA targets of the differentially regulated miRNAs, we used miRSystem, an integrated database for target gene prediction [26]. For the 29 differentially expressed miRNAs identified in our model, an average of 198 target genes was predicted for each individual miRNA. However, *in silico* miRNA target prediction results in high numbers of false-positives [27]. As multiple miRNA binding sites can cooperatively mediate post-transcriptional regulation of target gene expression [28], we hypothesized that genes targeted by multiple differentially expressed miRNAs are most likely to be genuine targets. To investigate this possibility, we used miRSystem to assess overlapping target genes predicted to be regulated by multiple members of the 29 differentially expressed miRNAs.

**Fig 2:**
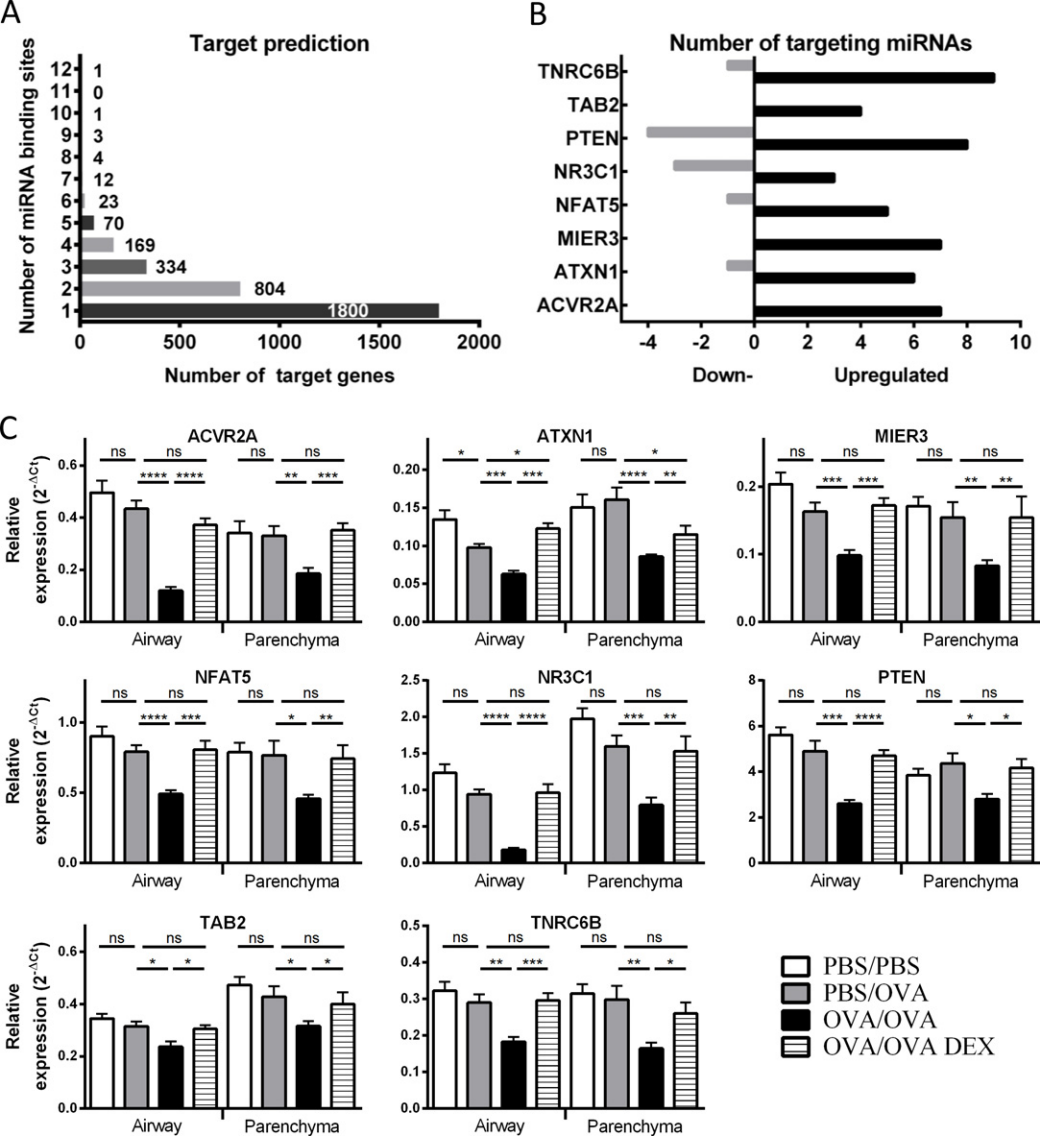
Predicted targets of altered miRNAs. Predicted target genes for the 29 differentially expressed miRNAs (based on miRSystem analysis) plotted against the number of predicted miRNA binding sites (A). Number of differentially expressed targeting miRNAs for 8 predicted target genes (change in OVA/OVA versus PBS/PBS) (B). qPCR assessment of 8 putative target genes in airway wall and parenchyma (C). Expression data is normalised relative to control HPRT. Error bars represent SEM (n = 6 animals/group). Mann-Whitney test was used to compare two groups with another. P values of less than 0.05 are indicated by *, less than 0.01 by **, less than 0.001 by *** and less than 0.0001 by ****.

Over 40% of the computated target genes (based on miRSystem) had predicted miRNA-binding sites for more than 1 of the 29 differentially regulated miRNAs, while ~20% had 3 or more predicted miRNA binding sites. A small number (44 target genes, representing just over 1% of all computated targets), had 6 or more predicted miRNA binding sites for the differentially expressed miRNAs (Fig 2A, Table 3). On average, the 44 candidate genes were targeted by 7 differentially expressed miRNAs each, of which 6 were upregulated and 1 was downregulated (Table 3).

**Table 3:** Predicted target genes with multiple target sites. Genes with target sites for 6 or more of the 29 identified differentially regulated miRNAs. The number of targeting miRNAs that are upregulated and downregulated are shown as well as the difference.

Target gene	Gene description	Number of targeting miRNAs	Upregulated miRNAs	Downregulated miRNAs	Difference
**PTEN**	phosphatase and tensin homolog	12	8	4	+4
**TNRC6B**	trinucleotide repeat containing 6b	10	9	1	+8
**CPEB3**	cytoplasmic polyadenylation element binding protein 3	9	7	2	+5
**TBL1XR1**	transducin (beta)-like 1X-linked receptor 1	9	7	2	+5
**AAK1**	AP2 associated kinase 1	9	8	1	+7
**SYNCRIP**	synaptotagmin binding, cytoplasmic RNA interacting protein	8	7	1	+6
**ACSL4**	acyl-CoA synthetase long-chain family member 4	8	7	1	+6
**CSNK1G1**	casein kinase 1, gamma 1	8	7	1	+6
**SERTAD2**	SERTA domain containing 2	8	7	1	+6
**ZFP148**	zinc finger protein 148	7	5	2	+3
**ATXN1**	ataxin 1	7	6	1	+5
**BCL2L2**	BCL2-like 2	7	5	2	+3
**ZFP704**	zinc finger protein 704	7	5	2	+3
**MBNL1**	muscleblind-like 1 (Drosophila)	7	6	1	+5
**TMEM55B**	transmembrane protein 55b	7	7	0	+7
**ELAVL2**	ELAV (embryonic lethal, abnormal vision, Drosophila)-like 2	7	4	3	+1
**RICTOR**	RPTOR independent companion of MTOR, complex 2	7	7	0	+7
**ACVR2A**	activin receptor IIA	7	7	0	+7
**NUFIP2**	nuclear fragile X mental retardation protein interacting protein 2	7	6	1	+5
**MIER3**	mesoderm induction early response 1, family member 3	7	7	0	+7
**MECP2**	methyl CpG binding protein 2	7	6	1	+5
**ZCCHC14**	zinc finger, CCHC domain containing 14	6	6	0	+6
**AFF4**	AF4/FMR2 family, member 4	6	4	2	+2
**TGFBR1**	transforming growth factor, beta receptor I	6	5	1	+4
**DCX**	doublecortin	6	5	1	+4
**CAMTA1**	calmodulin binding transcription activator 1	6	4	2	+2
**TCF4**	transcription factor 4	6	4	2	+2
**FBXO28**	F-box protein 28	6	5	1	+4
**SMG1**	SMG1 homolog, phosphatidylinositol 3-kinase-related kinase	6	6	0	+6
**RBM33**	RNA binding motif protein 33	6	4	2	+2
**FUBP1**	far upstream element (FUSE) binding protein 1	6	6	0	+6
**QK**	quaking	6	5	1	+4
**UBE2D3**	ubiquitin-conjugating enzyme E2D 3 (UBC4/5 homolog, yeast)	6	5	1	+4
**GSK3B**	glycogen synthase kinase 3 beta	6	5	1	+4
**PHF17**	PHD finger protein 17	6	5	1	+4
**IGF1R**	insulin-like growth factor I receptor	6	4	2	+2
**SOX6**	SRY-box containing gene 6	6	5	1	+4
**NR3C1**	nuclear receptor subfamily 3, group C, member 1	6	3	3	0
**NFAT5**	nuclear factor of activated T-cells 5	6	5	1	+4
**NF1**	neurofibromatosis 1	6	5	1	+4
**NAA50**	N(alpha)-acetyltransferase 50, NatE catalytic subunit	6	6	0	+6
**MLL1**	myeloid/lymphoid or mixed-lineage leukemia 1	6	4	2	+2
**SOX11**	SRY-box containing gene 11	6	4	2	+2
**TRIM2**	tripartite motif-containing 2	6	5	1	+4
	**Average**	7	6	1	+4

To determine whether our *in silico* approach predicted real changes in target mRNA expression levels, we characterised selected target genes in airway wall and parenchyma samples (Fig 2B and 2C). ACVR2A, MIER3, PTEN and TNRC6B have the highest number of putative binding sites for upregulated miRNAs (7 to 9) and ACVR2A, MIER3 and TNRC6B have the highest difference between up- and downregulated miRNAs (up to 8). The remaining 4 target genes were assessed based on previous literature: ATXN1 has been associated with asthma [29], NFAT5 is implicated in the regulation of proinflammatory cytokines [30], NR3C1 is a target for steroid-based asthma treatments [31] and TAB2 is a validated miR-155-5p target [32]. All 8 of the tested target genes were significantly downregulated in the OVA/OVA group in both airway wall and parenchyma and this difference was again sensitive to DEX treatment (Fig 2C). Taken together, our bioinformatic analysis identified novel asthma-associated genes that may be regulated by a network of differentially expressed miRNAs.

### Mir-155 is induced in allergic lung tissues and immune cells

**Fig 3:**
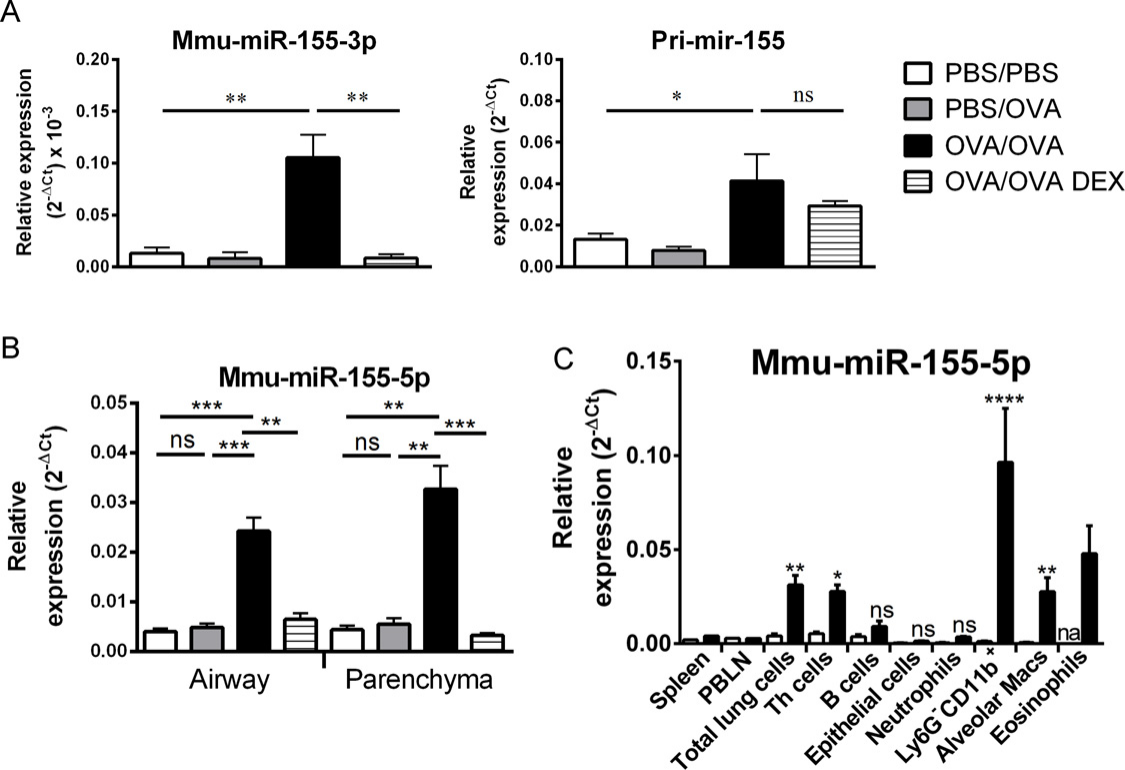
Mir-155 expression profiles in allergic lung tissues and isolated immune cells. qPCR assessment of expression levels for primary mir-155 and mature miR-155-3p in whole lungs from PBS/PBS, OVA/OVA and OVA/OVA DEX-treated mice (A). miR-155-5p expression levels assessed in the airway wall and parenchyma of PBS/PBS and OVA/OVA mice (B). miR-155-5p expression in spleen, PBLNs, total lung cells and FACS-sorted lung cell subsets (C). Th cells, B cells, epithelial cells, neutrophils, Ly6G^-^CD11b^+^ cells (predominantly DCs, macrophages and monocytes), alveolar macrophages (Macs) and eosinophils. See Table 4 for surface marker expression on the sorted cell populations. Expression data is normalised relative to sno202 for mature miRNAs and HPRT for primary mir-155. Error bars represent SEM for A and B (n = 8 animals/group). Mann-Whitney test was used to compare two groups with another. P values of less than 0.05 are indicated by * and less than 0.01 by ** and less than 0.001 by ***.

As miR-155-5p was the most differentially upregulated miRNA identified in our study (~5 fold-change in OVA/OVA versus control groups) and was reduced to baseline following steroid treatment, we chose miR-155-5p for further analysis. We detected increased levels of both pri-mir-155 and miR-155-3p transcripts in OVA/OVA mice (Fig 3A). The absolute expression level of miR-155-3p was much lower than miR-155-5p (Figs 1D and 3A). While miR-155-3p was reduced following DEX treatment, no change was detected in pri-mir-155 expression levels (Fig 3A).

We also performed experiments to assess which cell types express miR-155-5p in our model. In isolated airway wall and parenchymal tissue samples, similar expression levels of miR-155-5p were detected with significant increases in OVA/OVA mice (Fig 3B). There was no detectable change in miR-155-5p expression in the spleen or PBLN, although increased expression was confirmed in total lung cells (Fig 3C). In sorted lung cell populations (sorting strategy indicated in Table 4), increased miR-155-5p expression was detected in Th cells, Ly6G^-^CD11b^+^ cells (predominantly dendritic cell, macrophage and monocyte populations), and alveolar macrophages from OVA/OVA mice, compared to controls (Fig 3C). High miR-155-5p expression was also detected in eosinophils, with low expression observed in sorted epithelial cells and neutrophils (Fig 3C). These results clearly show that miR-155-5p is expressed by most haematopoietic cells in the lung and is upregulated in the context of allergic inflammation.

**Table 4:** Sorting strategy for different cell populations. Sorted cell populations were gated based on FSC (forward scatter), SSC (side scatter) and surface marker expression characteristics.

Identifier	Th cells	B cells	Epithelial cells	Neutrophils	Ly6G^-^CD11b^+^ cells	Alveolar Macs	Eosinophils
**FSC**	low	low		low	Int	high	low
**SSC**	low	low		Int	low	high	high
**CD45**	**+**	**+**	**-**	+	+	+	+
**B220**	-	**+**		Int	-	-	-
**CD4**	**+**	-		-	-	-	-
**CD8**	-	-		-	-	Int	-
**CD11b**	-	-		**+**	**+**	**+**	**+**
**CD11c**	-	-		Int	Int	**+**	**-**
**Epcam**			**+**				
**Ly6G**	-	-		**+**	**-**	Int	Int
**SiglecF**	-	-		-	-	**+**	**+**

### *In vivo* antagomir-based targeting of miR-155-5p decreases expression, but has no impact on AAD severity

**Fig 4:**
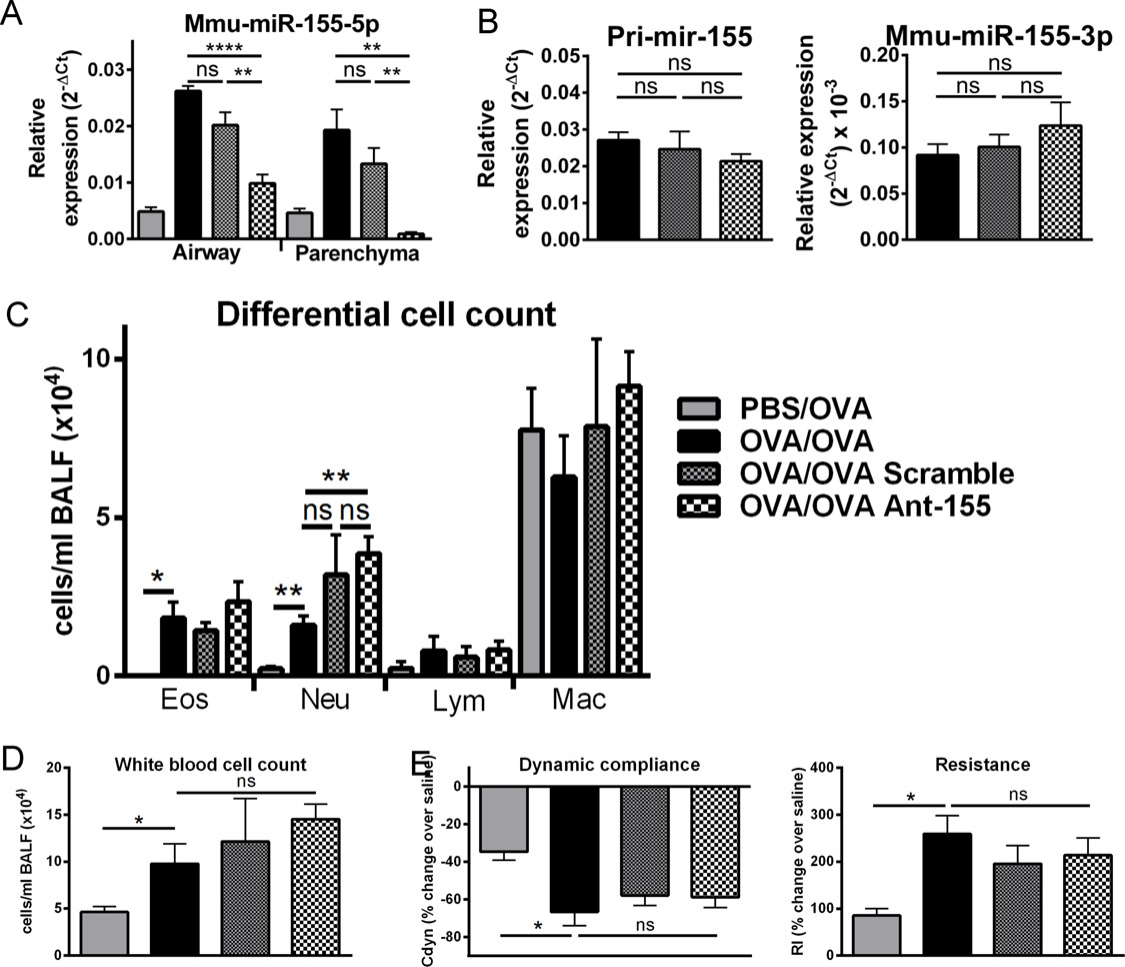
In vivo targeting of miR-155-5p with an antagomir effectively decreases expression but has no impact on disease. Expression of miR-155-5p in airway wall and parenchymal tissue samples of OVA/OVA mice treated with non-specific Scramble or miR-155-5p-specific Ant-155 (A). Primary mir-155 and mature miRNA miR-155-3p, in whole lungs of OVA/OVA mice treated with non-specific Scramble or miR-155-5p-specific Ant-155 (B). Expression data is normalised relative to sno202 for mature miRNAs and HPRT for primary mir-155. Eosinophil (Eos), neutrophil (Neu), lymphocyte (Lym) and macrophage (Mac) (C) and total cell numbers (D) in BALF from OVA/OVA mice treated with non-specific Scramble or Ant-155. Total lung resistance and dynamic compliance in response to 5 mg/ml inhaled methacholine in OVA/OVA mice treated with non-specific Scramble or Ant-155, presented as percentage change over baseline (saline) (E). Error bars represent SEM (n = 8 animals/group). Mann-Whitney test was used to compare two groups with another. P values of less than 0.05 are indicated by *, less than 0.01 by ** and less than 0.0001 by ****.

MiR-155-5p is highly upregulated in OVA/OVA mice, highly expressed in infiltrating immune cell populations and has previously been shown to play key roles in inflammation [6, 33–35]. We therefore sought to assess its function *in vivo* using an antagomir (Ant-155). Intranasal administration of Ant-155 significantly reduced miR-155-5p levels both in airway wall and parenchymal tissue samples (Fig 4A), with no effect on miR-155-3p or pri-mir-155 (Fig 4B), compared to scrambled antagomir (Scramble)-treated controls. Antagomir administration reduced miR-155-5p levels to the levels detected in PBS/OVA animals.

Having established that intranasal Ant-155 treatment effectively reduced miR-155-5p levels *in vivo*, we examined the effect of Ant treatment on hallmark features of AAD. Ant-155 had no significant effect on immune cell infiltration into the broncho-alveolar space following allergen challenge (Fig 4C and 4D) or the development of methacholine-induced AHR (Fig 4E and Figure A in S2 Fig). While Ant treatment alone (Scramble versus OVA/OVA control) showed a tendency towards increased BALF neutrophils, there were no changes in neutrophil numbers following specific Ant-155 treatment (Ant-155 versus Scramble) (Fig 4C). Ant-155 treatment also had no effect on gene expression for key genes induced in our AAD model (IL-5, IL-13 and Muc5ac; Figure B in S2 Fig). Ant-155 treatment did have a small impact on expression of two predicted target genes (ACVR2A and TAB2; Figure C in S2 Fig). Taken together, we show effective and specific inhibition of miR-155-5p following intranasal antagomir administration, but failed to detect any discernible effect on AAD severity.

### Antagomir uptake differs across immune cell populations *in vitro* and *in vivo*

**Fig 5:**
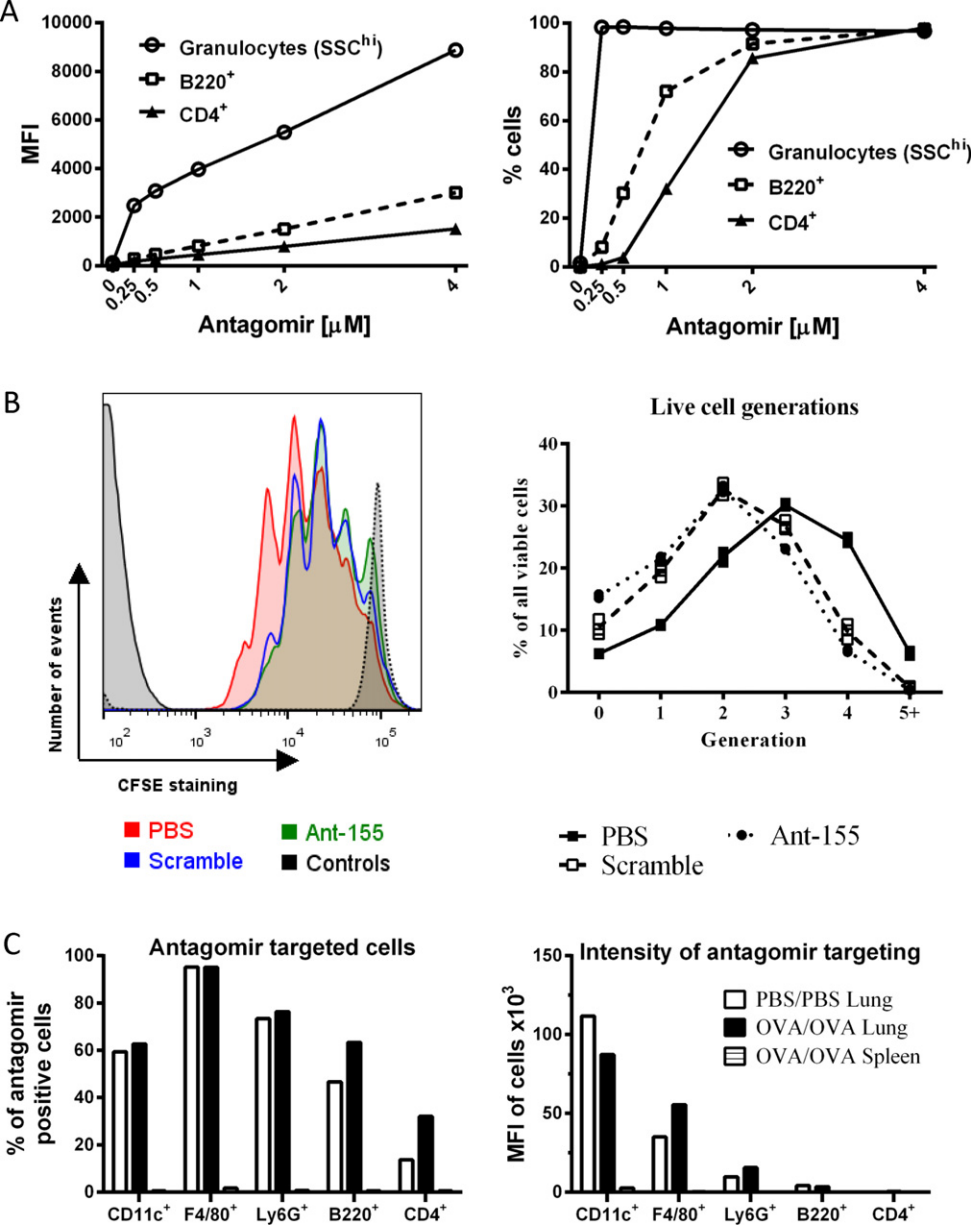
Antagomir efficiency varies across immune cell populations. Isolated lung cells were incubated with fluorescently (DY547)-labeled Ant at indicated concentrations for 1 hour. Mean fluorescent intensity and percentage of DY547^+^ cells for granulocytes (SSC^hi^), B220^+^ and CD4^+^ cells (A). CD4^+^ T cells were CFSE-labeled and cultured with 5 μM Ant for 3 days. Representative histogram plots and proliferation analysis for unlabeled and unstimulated, CFSE-labeled controls (Controls), PBS-treated (PBS) and Ant-treated (either Scramble or Ant-155) CD4^+^ T cells (B). Representative graphs from 3 independent experiments. Error bars SEM (n = 3 replicates/group). Fluorescently-labeled Ant uptake by lung immune cells *in vivo* following intranasal Ant administration (C). Mean fluorescent intensity and percentage of DY547^+^ cells for CD11c^+^, F4/80^+^, Ly6G^+^, B220^+^ and CD4^+^ cell populations.

Recent literature using mir-155^-/-^ mice suggests a functional role for miR-155-5p in AAD, in particular CD4 T cells [18, 19]. Based on these findings, it was surprising that we failed to detect a phenotype in our model, despite specific inhibition of miR-155-5p. To better understand our differing results, weassessed whether Ant uptake is altered across lung cell subsets using a fluorescent antagomir. Granulocytes (SSC^hi^) exhibited high levels of uptake across all Ant concentrations *in vitro*, with nearly 100% of cells positive at 0.25 μM Ant (Fig 5A). In contrast, B220^+^ and CD4^+^ cells, exhibited lower uptake at comparable concentrations (Fig 5A). For B220^+^ cells, 1 μM Ant was required to obtain >75% positive fluorescence (Fig 5A). For CD4^+^ cells, 2 μM fluorescent Ant were necessary to obtain >80% positively-labeled cells (Fig 5A). Even at concentrations of 4 μM Ant, CD4^+^ cells exhibited lower antagomir uptake (based on MFI) than granulocytes treated with 0.25 μM Ant (Fig 5A). At the high doses of Ant required for CD4^+^ cell uptake (5 μM), we noticed significant impacts on cell viability and proliferation. Exposure to either non-specific Scramble or miRNA-specific Ant-155 significantly reduced CD4^+^ T cell proliferation, with a clear reduction in the numbers of CD4^+^ T cells that have divided 4 or more times in a CFSE-based proliferation assay (Fig 5B). This effect was also observed with other sequence-specific Ant (data not shown). Taken together, these results demonstrate that effective antagomir uptake is both dose- and cell type-dependent. While myeloid cells can be targeted effectively at low doses, targeting of lymphocytes (CD4^+^ T cells and B220^+^ B cells) is less efficient and effective uptake requires high concentrations that induce toxicity and inhibit cell proliferation.

To determine whether similar differences in uptake occur *in vivo*, we also administered fluorescently-labeled Ant intranasally to either control or OVA-sensitized/challenged mice. Similar to our findings *in vitro*, Ant fluorescence levels were high in CD11c^+^, F4/80^+^ and Ly6G^+^ populations (representative of DCs, macrophages and neutrophils, respectively) (Fig 5C). However, MFI was particularly low across all lymphocyte populations (B220^+^ and CD4^+^ representative for B cells and T cells) and CD4^+^ cells in particular exhibited low percentages of fluorescent Ant-positive cells (Fig 5C). No fluorescence was detected in spleen immune cells, suggesting that intranasal administration of Ant remains limited to the lung (Fig 5C).

We have also assessed the uptake of fluorescent Ant in blood, liver and lung immune cells following intranasal administration in naïve mice. A single dose of 50 μg Ant resulted in lower uptake in blood and liver immune cells compared to the lung, both in terms of percentage of stained cells as well as fluorescent intensity (Figures A and B in S3 Fig). Similarly to the lung, we again observed lower percentages of fluorescent Ant-positive lymphocytes in the blood and liver. Noticeably, lung epithelial cells (CD45^-^Epcam^+^) also show good uptake of Ant (Figures C and D in S3 Fig). Taken together, we show that intranasal Ant delivery effectively targets myeloid and dendritic cell populations in the lung, but has limited ability to target lung lymphocytes and remains largely restricted to cells within the lung.

## Discussion

Current knowledge on the role of miRNAs in asthma pathogenesis remains limited. One previous study has used an acute OVA-induced model of AAD (aerosol delivery and challenge over a 7-day timecourse), but failed to identify alterations in miRNAs previously identified in other mouse models of AAD (including let-7b, miR-21 or miR-145) [36]. Our assessment identified 29 differentially expressed miRNAs, approximately 13% of all expressed miRNAs (228 total), comparable to the percentage of altered genes found in previous mRNA profiling studies [37–39]. The majority of the altered miRNAs (22 of 29) were increased in disease, suggesting a general upregulation of miRNA expression in our model of AAD.

Most of the differentially expressed miRNAs identified (26 of 29) were sensitive to DEX treatment. This is similar to data from asthmatic patient samples where the majority of differentially expressed miRNAs in airway epithelial cells are at least partially restored upon steroid treatment [9]. Steroids have an anti-inflammatory effect and may regulate miRNA expression in the lung either directly or indirectly (e.g. by decreasing lung inflammation and immune cell infiltration). The latter is particularly relevant for miRNAs expressed at high levels in inflammatory cells, such as miR-155-5p [40].

Interestingly, 5 of the 29 differentially expressed miRNAs identified in our study (let-7d-3p, miR-18a-5p, -21a-5p, -155-5p and -223-3p) have previously been implicated in inflammatory diseases [6, 14, 15, 18, 33, 41–45]. Furthermore, there is evidence for a contribution of let-7, mir-21 and mir-155 in AAD [12–15, 17–19]. Conflicting results have been reported for the role of let-7 in AAD, which may be explained by varying approaches employed to target let-7 expression (e.g. let-7 family inhibitor vs. let-7 mimic vs. let-7b specific inhibitor) as well as differences in the studied models (e.g. OVA vs. HDM) [14, 15, 17].

Based on the identified differentially expressed miRNAs, we performed computational predictions of miRNA-gene interactions to identify potential downstream targets. To limit prediction variability, we used miRSystem, which requires at least three different algorithms to predict the same miRNA-target gene interaction and importantly, also includes experimentally verified targets [26]. To provide further stringency, we focused on genes predicted to be targeted by more than one of the differentially expressed miRNAs. This approach identified 44 genes that were predicted to be targeted by at least 6 of the 29 identified miRNAs.

From the list of 44 predicted target genes, we analysed the expression of 8 genes (ACVR2A, ATXN1, MIER3, NFAT5, NR3C1, PTEN, TAB2 and TNRC6B). As predicted, all 8 target genes were significantly downregulated in OVA/OVA mice. Importantly, our analysis demonstrated changes in all tested targets, contrary to the high false-positive rates that typically occur using *in silico* target prediction [27]. To date, *in silico* target prediction rarely accounts for multiple miRNA binding sites in predicted target genes, despite evidence suggesting that multiple miRNA binding sites per target gene are common and the potential for synergistic regulation of miRNA target genes [46–50]. As such, we suggest that using combinatorial *in silico* target prediction may be useful at identifying true targets in other miRNA profiling studies.

Three of the predicted target genes, namely ATXN1, NR3C1 and PTEN, have previously been associated with asthma [29, 31, 51–57]. ATXN1 regulates extracellular matrix remodeling and lung alveolarization [29]. ATXN1 was significantly reduced in OVA/OVA mice, suggesting that miRNA-mediated inhibition of ATXN1 may contribute to remodeling in this model of AAD. NR3C1, also known as glucocorticoid receptor (GR), binds to GR-response elements (GRE) and activates transcription of GRE-controlled genes [31, 58]. GR translocation suppresses inflammatory gene expression and glucocorticoids are a main-stay treatment in severe asthmatic disease [31]. Of the 6 differentially regulated miRNA predicted to target NR3C1, two (miR-18a and miR-142-3p) have previously been shown to target NR3C1 directly [59–63]. While PTEN is best characterised as a cancer-related gene, it is also proposed to regulate of asthma pathogenesis [54–57, 64, 65]. PTEN protein levels are reduced in the lungs of allergen challenged mice, which correlates with increased PI3K activity [55].

The remaining predicted target genes (ACVR2A, MIER3, NFAT5, TAB2 and TNRC6B) are poorly studied. ACVR2A is a receptor kinase in the activin signaling pathway that regulates proliferation and is directly targeted by miR-29b and miR-181a [66–68]. MIER3 is a protein with no known function. NFAT5 is a member of the NFAT/Rel family of transcription factors [69] that regulates the production of proinflammatory cytokines [30]. TAB2 is targeted by miR-23b and miR-155 [32, 70] and while no role for TAB2 has been identified in asthma, it is an adaptor in the TLR/IL-1 signaling cascade and may regulate inflammation in asthma [32]. TNRC6B associates with Argonaute proteins and promotes miRNA target gene regulation [71–73]. The finding that TNRC6B may also be regulated by miRNAs, suggests a potential feedback loop whereby altered miRNA expression may impact on miRNA-mediated regulation processes in AAD.

Our findings provide novel insight into the complex regulatory networks underlying AAD and identify several differentially expressed target genes not previously associated with asthma, which may provide insights into disease mechanisms. More broadly, our findings highlight the impact of multiple altered miRNAs on individual genes and the utility of considering overlapping miRNA expression to better identify targets. It remains to be determined experimentally whether our identified target genes are directly targeted by individual miRNAs. Furthermore, our target prediction is based on samples from whole lung and its interpretation is complicated due to significant changes to the cellular composition in OVA/OVA lungs. Increased expression of miR-155-5p in Th cells, DC and macrophage populations as well as high expression in infiltrating eosinophils in OVA/OVA mice is likely to contribute to the observed increases in total lung. Similarly, predicted target gene expression may be altered by both altered expression levels in lung cells as well as the influx of immune cells. Further experiments are necessary to determine how miRNAs regulate these *in silico* identified target genes, whether they are direct miRNA targets and whether they contribute directly to asthma pathogenesis.

MiR-155-5p was the most differentially regulated miRNA identified in our study (~5 fold-change in OVA/OVA versus control groups), was sensitive to steroid treatment and highly expressed in infiltrating eosinophils and increased in Th cell, DC and macrophage populations in the lung. MiR-155-5p is increased in hematopoietic cells following activation of B cells, T cells, macrophages and DCs [41, 74, 75] and has been implicated in various inflammatory diseases, including rheumatoid arthritis, multiple sclerosis, inflammatory bowel disease and atopic dermatitis [33, 35, 76–78]. MiR-155 is also increased in the nasal mucosa of allergic patients with asthma [10] and airway smooth muscle cells from asthmatic patients [11]. Recently, mir-155^-/-^ mice (which lack pri-mir-155, miR-155-5p and miR-155-3p) were shown to have decreased lung inflammation in models of AAD [18, 19]. However, the protective effect was not complete and the impact on lung function and lung remodeling was not assessed. The latter is of interest, as mir-155^-/-^ mice develop spontaneous lung remodeling with age [79]. Based on these previous studies, we assessed whether mature miR-155-5p plays a role in our model and whether inhibitor-based treatment could alter disease pathology.

Although the exact mechanism of Ant uptake into cells is unknown, work by Krutzfeldt *et al*. has shown that the chemical properties of antagomirs (increased lipophilicity due to a cholesterol tag at the 3’-end) are responsible for the cellular uptake into cells [80]. Systemic Ant-mediated targeting of miRNA function *in vivo* results in effective, specific, long-lasting silencing in many tissues [80, 81]. We and others have previously established that intranasal administration of Ant effectively inhibits miRNA function in the lung [16, 17, 20, 82]. Intranasal Ant-155 administration resulted in specific inhibition of miR-155-5p in both the airway wall and parenchymal tissue samples. This was accompanied by slight increases in two predicted targets of miR-155-5p (ACVR2A and TAB2) although expression remained below levels in control animals. Three other predicted targets (MIER3, NFAT5 and TNRC6B) were not altered following Ant-155 administration. The overall efficacy of Ant-mediated knockdown of miR-155-5p was incomplete and comparable to levels detected in PBS/OVA animals and had no discernible therapeutic effect on hallmark features of disease such as AHR, inflammation or mucus production. Importantly, neither pri-mir-155 nor miR-155-3p expression levels were altered following Ant-155 delivery, suggesting highly specific targeting of mature miR-155-5p. This is different from other studies that have targeted the mir-155 gene using knockout mice or precursor molecules, which alter 5p and 3p mature miRNAs and the precursor miRNA (pri-miR-155) systemically [18, 19, 33, 79, 83]. These differences in experimental approach may explain why we failed to observe any impact of Ant-155 administration on AAD.

Antagomir studies have mainly investigated whole tissue rather than investigating specific cell types [16, 17, 81, 84–86]. Little is known about the pharmacokinetics and distribution of Ant uptake following intranasal administration of Ant. *In vitro*, administered Ant was quickly internalized by cells but uptake efficiency varied greatly across different cell types. Myeloid cells (including macrophages and neutrophils) exhibited higher levels of Ant uptake compared to lymphocytes. Similarly, *in vivo*, myeloid cells in the lung exhibited high levels of fluorescent Ant uptake, while lymphocytes, in particular T cells, exhibit poor uptake. This cellular specificity was also observed in the blood and liver. Furthermore, immune cells in the blood and liver displayed much lower overall Ant uptake compared to lung immune cells, highlighting the tissue-specificity of the intranasal delivery route. We also observed efficient Ant uptake in lung epithelial cells, which expressed miR-155-5p at very low levels and showed no altered expression in our AAD model. This suggests that miR-155-5p in epithelial cells may not play a functional role in AAD. The differential Ant uptake across different lung cell types may explain why we failed to achieve a complete reduction in miR-155-5p with Ant-155 treatment.

Variability in Ant-mediated cell targeting could also explain the differences between our study and the partial protection from AAD observed in mir-155^-/-^ mice [18, 19]. Specifically, this difference may be driven by a functional role for miR-155-5p in lymphocyte function, which would be altered in mir-155^-/-^ mice, but not following Ant-155 administration. This hypothesis is supported by many studies showing a role for mir-155 in lymphocyte function and disease pathogenesis in bacterial and viral infections and lymphocyte-driven diseases such as EAE and RA in mir-155^-/-^ mice [79, 87–93]. We therefore speculate that the specific targeting of miR-155-5p in lymphocytes, in particular T cells may have a positive therapeutic benefit in the studied model of AAD. Recently, this has been shown to be the case in a HDM model of AAD, where mixed bone-marrow chimeras with mir-155-deficient T cells were partially protected from developing allergic airway inflammation and directly targeting mir-155 in Th2 cells prevented the development of Th2-mediated AAD [19]. However, the protective effect was not as pronounced as in mir-155^-/-^ mice, suggesting that other cell types also contribute to a protected phenotype. Furthermore, Okoye *et*.*al*. identified that T cell-intrinsic mir-155 was required for efficient Th cell recruitment to the lungs in this model of AAD [19]. In agreement with these findings, Malmhall *et*.*al*. also provide evidence for deficient Th2 cell differentiation and reduced numbers of lung Th cells in mir-155^-/-^ mice subject to a mouse model of AAD [18]. In contrast to mir-155^-/-^ mice, specific targeting of miR-155-5p in the lung with intranasal Ant delivery would not be able to prevent the recruitment of Th cells to the lung. Importantly, the use of mir-155^-/-^ mice does not answer the question whether mir-155 function is required during sensitization, challenge or both phases of AAD development. Taken together, these findings suggest that mir-155 function in T cells is largely responsible for the protective phenotype that is observed for mir-155^-/-^ mice in models of AAD.

## Summary

The current study provides insight into alterations in miRNA expression and their potential role in the pathogenesis of allergic asthma, using a mouse model of AAD. We successfully characterised the mouse lung miRNAome and identify novel gene changes following allergen challenge. While targeting of miR-155-5p with a specific antagomir resulted in specific inhibition *in vivo*, it failed to alter the disease phenotype. We propose that this lack of protection may result from the observed variation in antagomir uptake, with efficient uptake in myeloid cell populations and poor uptake in lymphocytes. Taken together, this study provides novel insight into the complex molecular processes underlying allergic asthma and the limitations of targeting miRNAs with antagomir-based strategies. The implications of our results also have important consequences on the interpretation of study results using Ant-based treatments.

## Supporting Information

S1 Fig Characterisation of the murine model of allergic airways disease. Total lung resistance and dynamic compliance (percentage change over baseline (saline)) in response to inhaled methacholine in PBS/OVA, OVA/OVA and OVA/OVA DEX-treated mice (A). Total BALF white blood cell and differential eosinophil (Eos), neutrophil (Neu), lymphocyte (Lym) and macrophage (Mac) counts (B). Mucus secreting cell (MSC) and eosinophil counts per 100μm high power field in lung tissue sections (C). IL-5, IL-13 and Muc5ac mRNA expression levels in airway wall and parenchyma by qPCR (D). Expression data normalised to housekeeping HPRT control RNA levels. Error bars represent SEM (n = 6 animals/group). Mann-Whitney test was used to compare two groups with another. P values of less than 0.05 are indicated by *, less than 0.01 by **, less than 0.001 by *** and less than 0.0001 by ****.
(TIF)

S2 Fig Ant-155 administration has no effect on AHR and Th2 or target mRNA expression. Total lung resistance and dynamic compliance (percentage change over baseline (saline)) in response to inhaled methacholine in OVA/OVA treated with non-specific Scramble or miR-155-5p-specific Ant-155 (A). IL-5, IL-13 and Muc5ac mRNA expression levels in airway wall and parenchyma tissue by qPCR (B). ACVR2A, MIER3, NFAT5, NR3C1, PTEN, TAB2 and TNRC6B mRNA expression levels in whole lung tissue samples by qPCR (C). Expression data is normalized to control RNA HPRT. Error bars represent SEM (n = 8 animals/group). Mann-Whitney test was used to compare two groups with another. P values of less than 0.05 are indicated by *.
(TIF)

S3 Fig Ant uptake in blood, liver and lung. Ant uptake by blood, liver and lung cells following *in vivo* Ant administration. 50 μg of DY547-labeled Ant was administered intranasally and uptake determined in single cell preparations from blood, liver and lung after 24 h. Percentage of DY547^+^ cells of CD4^+^, B220^+^ and CD11b^+^ cell populations is shown (A). Mean fluorescent intensity of CD4^+^, B220^+^ and CD11b^+^ cell populations is shown (B). Percentage of DY547^+^ (C) and mean fluorescent intensity (D) of lung epithelial cells (CD45^-^Epcam^+^) in the lung is shown. Error bars represent SEM (n = 3). Mann-Whitney test was used to compare two groups with another. P values of less than 0.05 are indicated by *.
(TIF)
