# Supplementary figures and images for: MicroRNA Expression Is Altered in an Ovalbumin-Induced Asthma Model and Targeting miR-155 with Antagomirs Reveals Cellular Specificity
Source: PLoS One. 2015 Dec 22;10(12):e0144810. doi: 10.1371/journal.pone.0144810 (PMC4691205; doi:10.1371/journal.pone.0144810)

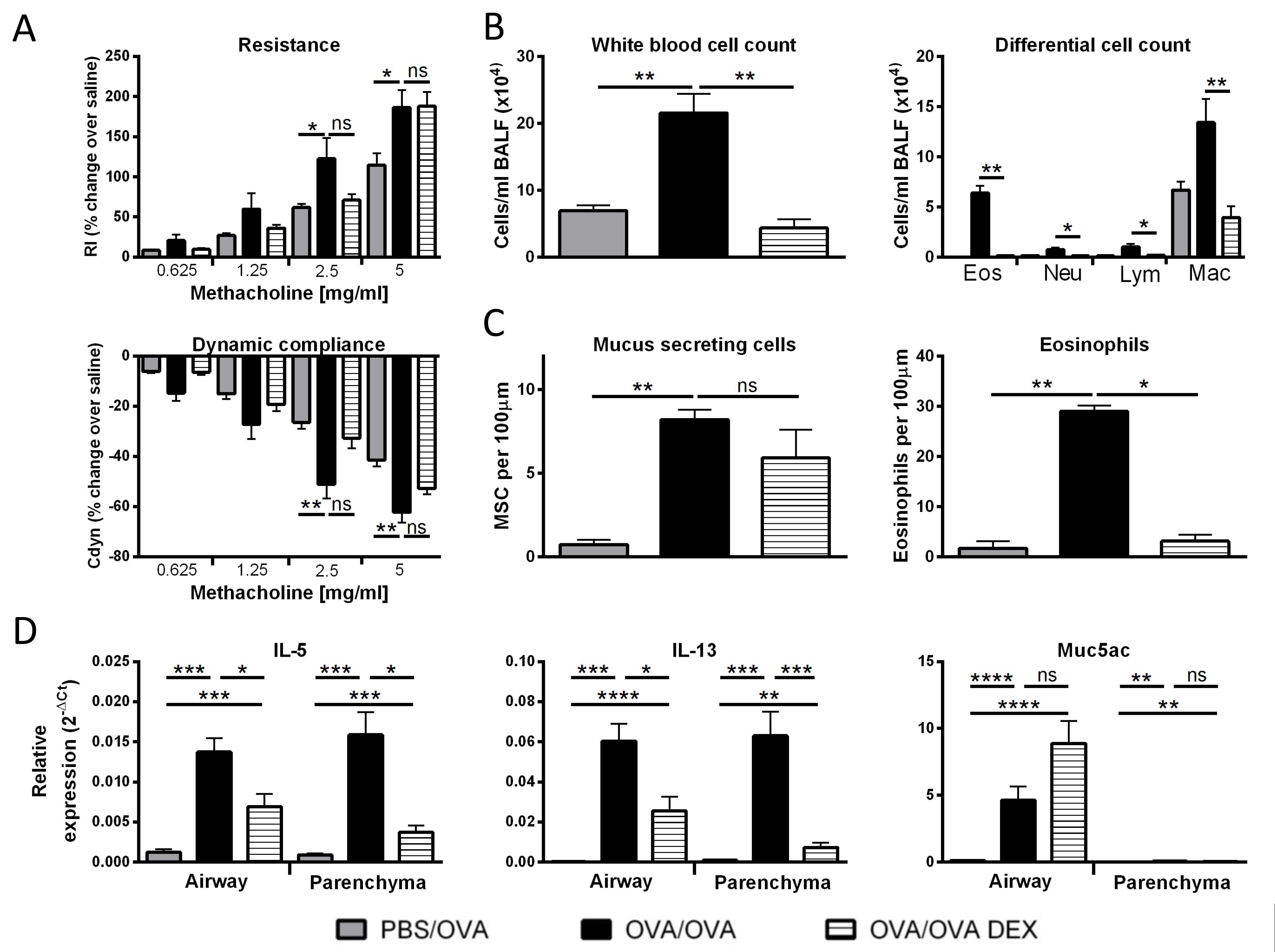

Supplement: S1 Fig — Total lung resistance and dynamic compliance (percentage change over baseline (saline)) in response to inhaled methacholine in PBS/OVA, OVA/OVA and OVA/OVA DEX-treated mice (A). Total BALF white blood cell and differential eosinophil (Eos), neutrophil (Neu), lymphocyte (Lym) and macrophage (Mac) counts (B). Mucus secreting cell (MSC) and eosinophil counts per 100μm high power field in lung tissue sections (C). IL-5, IL-13 and Muc5ac mRNA expression levels in airway wall and parenchyma by qPCR (D). Expression data normalised to housekeeping HPRT control RNA levels. Error bars represent SEM (n = 6 animals/group). Mann-Whitney test was used to compare two groups with another. P values of less than 0.05 are indicated by *, less than 0.01 by **, less than 0.001 by *** and less than 0.0001 by ****. (TIF) [file pone.0144810.s001.tif]

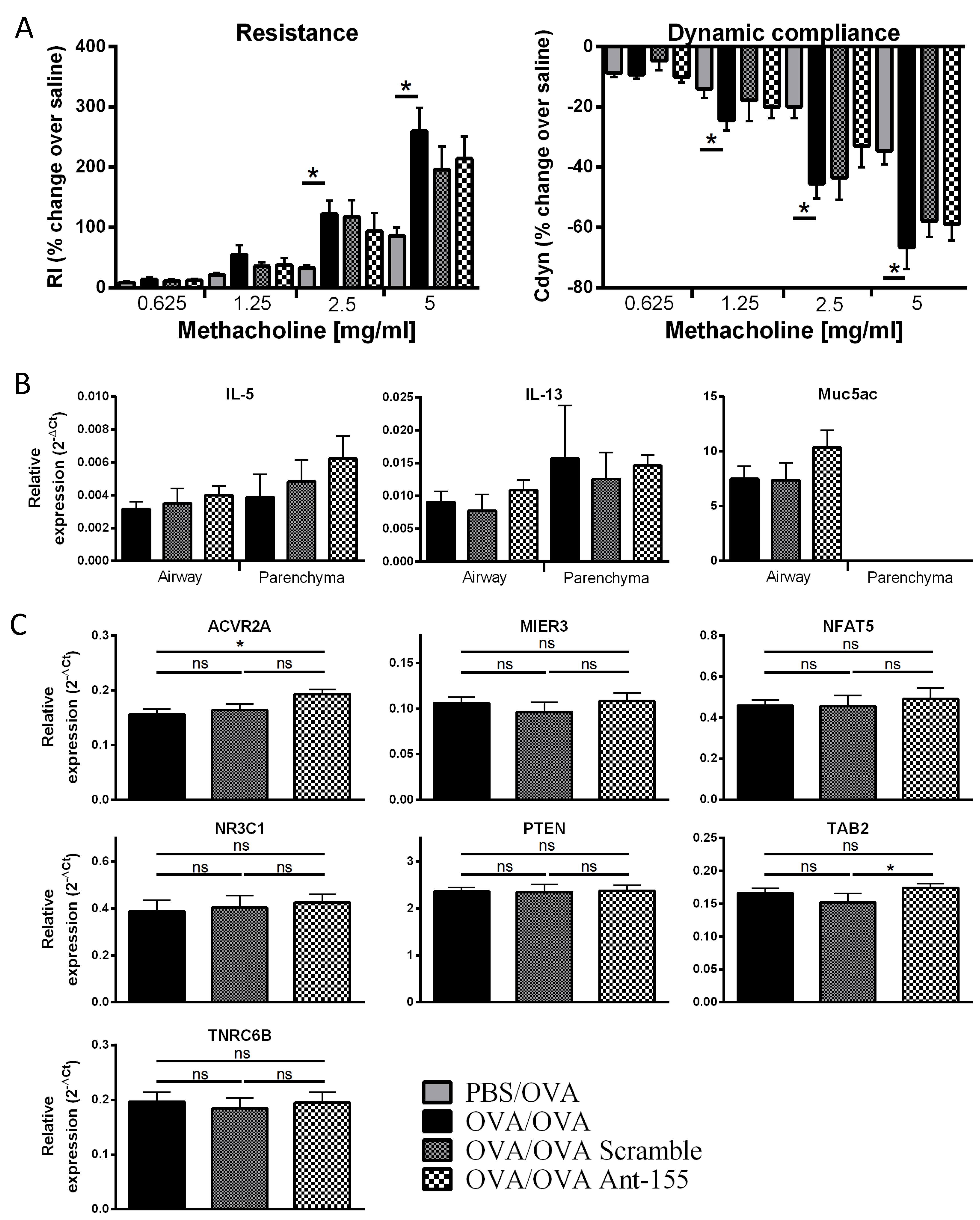

Supplement: S2 Fig — Total lung resistance and dynamic compliance (percentage change over baseline (saline)) in response to inhaled methacholine in OVA/OVA treated with non-specific Scramble or miR-155-5p-specific Ant-155 (A). IL-5, IL-13 and Muc5ac mRNA expression levels in airway wall and parenchyma tissue by qPCR (B). ACVR2A, MIER3, NFAT5, NR3C1, PTEN, TAB2 and TNRC6B mRNA expression levels in whole lung tissue samples by qPCR (C). Expression data is normalized to control RNA HPRT. Error bars represent SEM (n = 8 animals/group). Mann-Whitney test was used to compare two groups with another. P values of less than 0.05 are indicated by *. (TIF) [file pone.0144810.s002.tif]

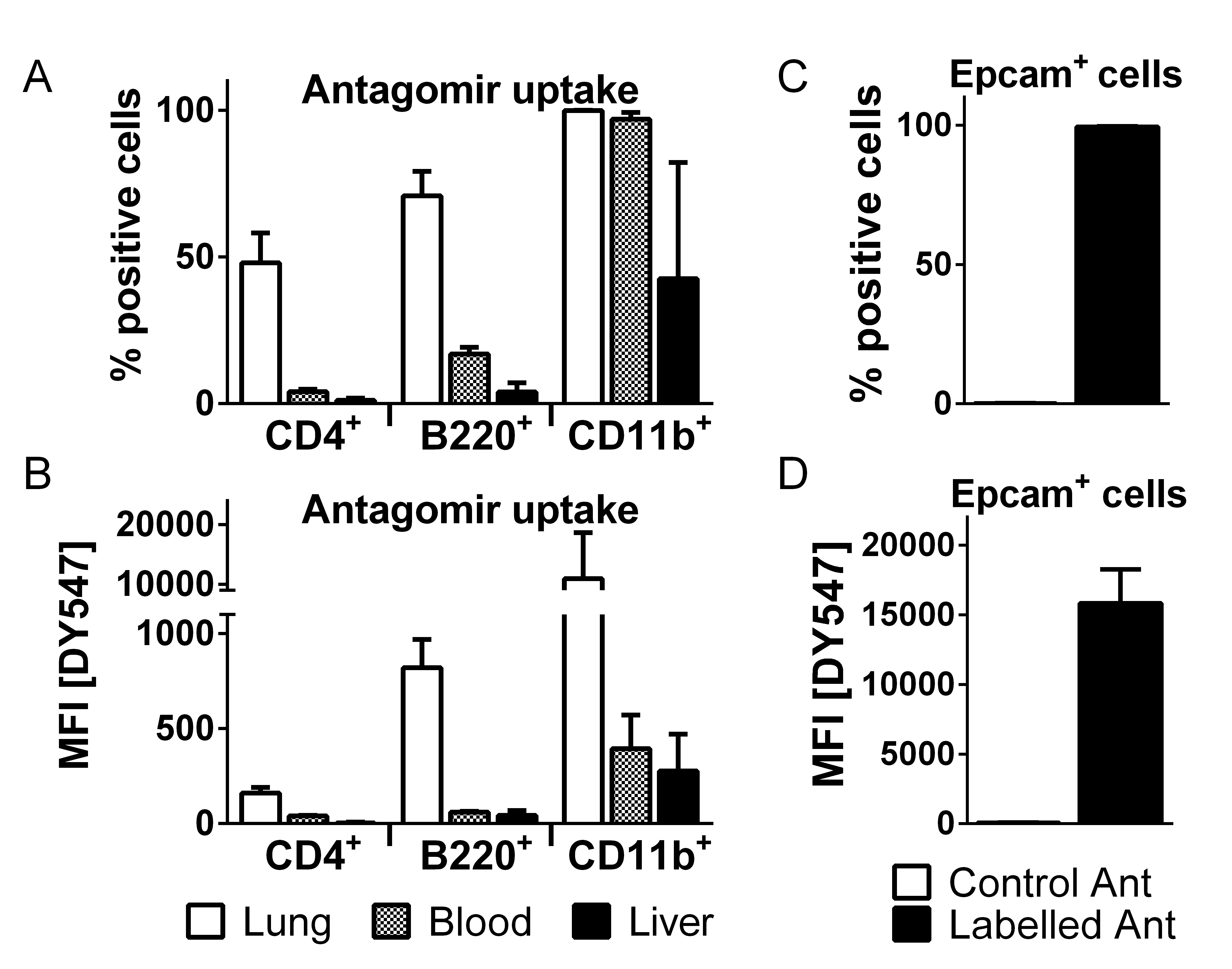

Supplement: S3 Fig — Ant uptake by blood, liver and lung cells following in vivo Ant administration. 50 μg of DY547-labeled Ant was administered intranasally and uptake determined in single cell preparations from blood, liver and lung after 24 h. Percentage of DY547+ cells of CD4+, B220+ and CD11b+ cell populations is shown (A). Mean fluorescent intensity of CD4+, B220+ and CD11b+ cell populations is shown (B). Percentage of DY547+ (C) and mean fluorescent intensity (D) of lung epithelial cells (CD45-Epcam+) in the lung is shown. Error bars represent SEM (n = 3). Mann-Whitney test was used to compare two groups with another. P values of less than 0.05 are indicated by *. (TIF) [file pone.0144810.s003.tif]
